# Photothermal hydrogel platform for prevention of post-surgical tumor recurrence and improving breast reconstruction

**DOI:** 10.1186/s12951-021-01041-w

**Published:** 2021-10-07

**Authors:** Xi Yang, Ling Gao, Yuanfeng Wei, Bowen Tan, Yongzhi Wu, Cheng Yi, Jinfeng Liao

**Affiliations:** 1 Department of Medical Oncology, Cancer Center, West China Hospital, Sichuan University, Chengdu, 610041 China; 2 State Key Laboratory of Oral Diseases, National Clinical Research Centre for Oral Diseases, West China Hospital of Stomatology, Sichuan University, Chengdu, 610041 China; 3 Department of Health Ward, The Affiliated Cancer Hospital of Guangzhou Medical University, Guangzhou, 510095 China

**Keywords:** Breast cancer, Hydrogel, Microspheres, Photothermal therapy, Breast reconstruction

## Abstract

**Background:**

As one of the leading threats for health among women worldwide, breast cancer has high morbidity and mortality. Surgical resection is the major clinical intervention for primary breast tumor, nevertheless high local recurrence risk and breast tissue defect remain two main clinical dilemmas, seriously affecting survival and quality of life of patients.

**Experimental:**

We developed a thermoresponsive and injectable hybrid hydrogel platform (IR820/Mgel) by integration of co-loaded porous microspheres (MPs) and IR820 for preventing postoperative recurrence of breast cancer via photothermal therapy and promoting subsequent breast reconstruction.

**Results:**

Our results suggested that IR820/Mgel could quickly heated to more than 50.0 ℃ under NIR irradiation, enabling killing effect on 4T1 cells in vitro and prevention effect on post-surgical tumor recurrence in vivo. In addition, the hydrogel platform was promising for its minimal invasion and capability of filling irregularly shaped defects after surgery, and the encapsulated MPs could help to increase the strength of gel to realize a long-term in situ function in vivo, and promoted the attachment and anchorage property of normal breast cells and adipose stem cells.

**Conclusions:**

This photothermal hydrogel platform provides a practice paradigm for preventing locally recurrence of breast cancer and a potential option for reconstruction of breast defects.

**Graphic abstract:**

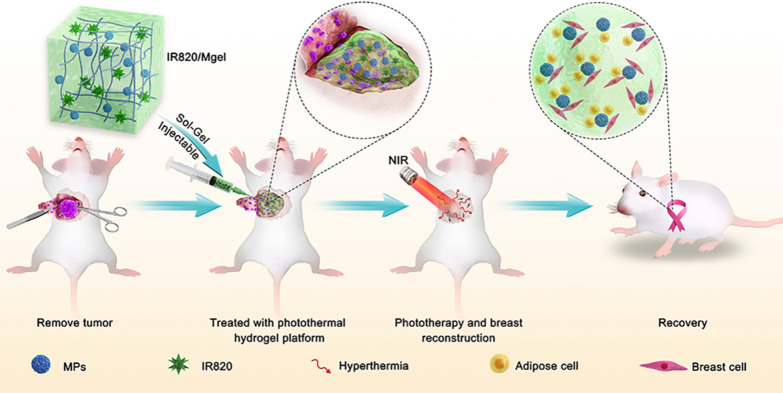

**Supplementary Information:**

The online version contains supplementary material available at 10.1186/s12951-021-01041-w.

## Introduction

Breast cancer is the most common malignancy in women worldwide [1]. In the United States, 279,100 new breast cancer cases and 42,690 breast cancer deaths are projected to occur in 2020 [2]. In the clinic, surgical resection is the major form of intervention strategy for primary breast cancer. Unfortunately, the local recurrence risk of breast cancer after surgery with a ranging from 10% to 41% remains a clinically fatal problem [3]. In addition, there are still some burdens of cancer patients with sexual dysfunction, loss of femininity, and psychological distress, which may result from breast defects [4]. Currently, breast reconstruction strategies including an implant-based procedure or an autologous-tissue flap are commonly used to repair breast imperfection [5]. However, these approaches are associated with many drawbacks such as capsular contracture, implant migration, extrusion and rupture, infection, and rippling [6]. Therefore, how to achieve breast reconstruction after surgery, as well as the effective suppression of tumor recurrence is the key problem to be solved.

Hydrogels as the postoperative fillers are widely studied for applications in breast reconstruction due to the demands of post-surgical patients [5, 7–9]. Both nature hydrogel and synthetic hydrogel are used to reconstruct breast. Synthetic hydrogel based on the synthetic polymers have been performed to the postoperative fillers in breast cancer patients, whereas their application for this purpose is limited by their inflammatory and potential toxic by-products of degradation [7, 10]. Currently, a broad diversity of nature hydrogel has been developed as a soft filler, such as collagen, hyaluronic acid, fibrin, methylcellulose and chitosan [11–14]. Nature hydrogel displays favorable advantages in soft reconstruction including similar molecular properties to extracellular matrix, biocompatibility, suitable mechanical properties, and low inflammation [15, 16]. In particular, thermoresponsive and injectable nature hydrogel has been expanded to an ideal breast filler due to their merits for minimally invasive, cost-effectiveness, and easy to operate, which could be dramatically molded into the breast cavity for matching the shape well. Among them, methylcellulose is actually a thermo-reversible nature hydrogel, which could form a physical cross-linking hydrogel simply by aggregation between the chains which can avoid chemical modification or use of crosslinking agents, making it favorable to prepare in situ injectable hydrogel [17]. However, these nature hydrogels with the numerous pores are not suitable for cell attachment due to the smooth surface of the pores, which is essential for tissue engineering. Biocompatible and biodegradable polymers that could offer a good support for cells attachment and anchorage are commonly used for tissue repairing, such as poly(lactic-coglycolic) acid (PLGA) and poly(*ε*-caprolactone)-*b*-poly(ethylene glycol)-*b*-poly(*ε*-caprolactone) (PECE) [18–20]. In our previous study, we have confirmed that polymer microspheres with porous structure and a rough surface could provide more anchor points for attaching of fibroblasts [21–23]. Therefore, it may be a promising option for breast reconstruction through developing the porous microspheres (MPs) loaded nature hydrogel with biocompatibility and low inflammation.

Compared with chemotherapy and radiotherapy, photothermal therapy (PTT) has attracted considerable attention due to its minimal invasiveness for breast cancer treatment [9, 24–28]. PTT as a noninvasive approach utilizes near-infrared (NIR) light-absorbing materials that can transform light to heat to remove tumor tissues through thermal ablation [29–31]. Currently, numerous PTT agents have been reported for cancer therapy, such as graphene, gold nanostructures, carbon dots, and NIR-absorbing organic dyes [31–34]. Among them, indocyanine green (ICG) is a NIR dye approved by the United States Food and Drug Administration (FDA) [35]. Currently, ICG is widely developed for tumor diagnosis and treatments by virtue of fluorescent properties [36, 37]. However, ICG displays some limitations as a pharmaceutical product, including low photostability, potential toxicity, and poor aqueous stability [38]. Despite a new indocyanine green (IR820) with biocompatibility improvement, the potential application of it in cancer therapy is restricted owing to rapid blood clearance and inability to specifically accumulate in target site [29]. To surpass these limitations, a hydrogel platform for local cancer treatment is an effective strategy.

**Scheme 1:**
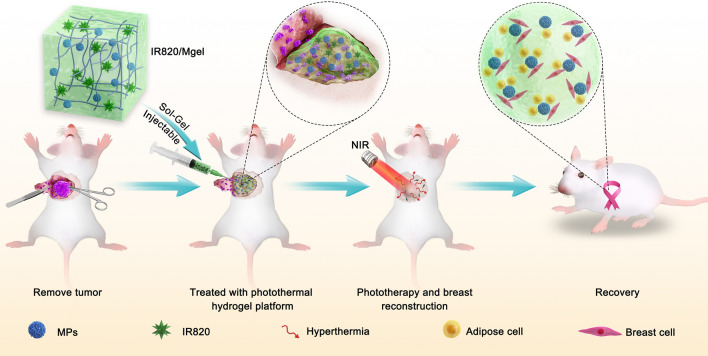
The scheme of hydrogel platform (IR820/Mgel) preparation and its applications for preventing post-surgical tumor recurrence and improving breast reconstruction. The IR820/Mgel helps forward IR820, as a photothermal agent retention in tumor site, thus presents an excellent photothemal performance for preventing locally recurrence of cancer. In addition, the hydrogel platform as a filler could repair the breast defects, at the same time porous MPs offer a well support for cells attachment and anchorage, which may provide benefits for breast reconstruction

Herein, as shown in Scheme 1, a thermoresponsive and injectable methylcellulose hydrogel platform (IR820/Mgel) loaded with IR820 and PLGA MPs was fabricated. The hybrid hydrogel platform could help retention of IR820 in the tumor bed, and act as a filler into the breast cavity. IR820, as a photothermal agent presented good photothermal performance in the tumor resection bed. MPs were loaded in the gel, which could help to increase the strength of gel to realize a long-term in situ function. The porous structure of MPs also provided more anchor points to long-term support the attachment of normal breast cells and adipose cells to facilitate breast reconstruction. The photothermal therapeutic ability of IR820/Mgel for preventing post-surgical breast cancer recurrence was studied by orthotopic breast tumor model. Further, the cell attachment and anchorage property of MPs and in vivo shaping and filling effect were explored to investigate the breast reconstruction potentiality of the hydrogel platform. Ultimately, this bifunctional hydrogel platform could be used as a promising treatment for preventing locally recurrence of breast cancer and a breast tissue reconstruction scaffold.

## Materials and methods

### Materials, cells and animals

Methylcellulose (with a viscosity of 15cP), PLGA (lactide/glycolide = 75/25, Mw = 22,000 Da), IR820 (Zhejiang Hisun Pharmaceutical Company), 3-(4, 5-dimethylthiahiazol-2-yl)-2, 5-diphenyl-2*H-*tetra-zolium bromide (MTT) were obtained from Sigma-Aldrich Chemicals (St. Louis, MO, USA). Live & Dead Viability/Cytotoxicity Assay Kit was purchased from Nanjing KeyGen Biotech Co., Ltd. (Nanjing, China). The anti-Ki67 rabbit polyclonal antibody and horseradish peroxidase-labeled goat anti-rabbit IgG antibody were purchased from Wuhan Servicebio Biological Technology Co., Ltd. (Wuhan, China).

Two mouse fibroblasts (L929 and NIH3T3 cells) and 4T1 breast cancer cells were obtained from the American Type Culture Collection (ATCC, USA). NIH3T3 and L929 were cultured in Dulbecco’s Modified Eagle’s Medium (DMEM, Gibco) containing 1% penicillin/streptomycin (Hyclone), and 10% fetal bovine serum (FBS, Gibco). Human breast epithelial cells (MCF-10A), a gift from Yanchu Li, Sichuan University were cultured in Dulbecco’s Modified Eagle Medium/Nutrient Mixture F-12 (DMEM/F12, Gibco) supplemented with 5% horse serum (HS, Gibco), 1% penicillin/streptomycin (Hyclone), 0.5 mg/mL hydrocortisone (Sigma), 20 ng/mL epidermal growth factor (EGF, Sigma), 10 μg/mL insulin (Sigma) and 100 ng/mL cholera toxin (Sigma). Adipose stem cells (a gift from Tao Zhang, Sichuan University) were cultured in α-MEM (Gibco) supplemented with 1% penicillin/streptomycin (Hyclone) and 10% FBS (Gibco). All cell lines were maintained at 37 ℃ in a humidified atmosphere 5% CO_2_.

BALB/c mice (Female, 6–8 weeks) were purchased from the Beijing Huafukang Biotechnology Co., Ltd. (Beijing, China) and housed in a pathogen-free conditions in a 12 h light–dark cycles with relative humidity of 50%–60% and constant room temperature (20 ± 1 ℃). All mice were adapted for 7 days before treatment. The animal procedures were performed in compliance with the Institutional Animal Care and Ethics Committee of Sichuan University (Chengdu, China).

### Preparation and characterization of PLGA porous MPs

The preparation of PLGA porous microspheres was based on a double-emulsion method. Briefly, 0.5% PVA solution was prepared by stirring in a 60–70 ℃ water bath, and 3.75 mL of the PVA solution was taken to dissolve NH_4_HCO_3_ (5%) to prepare a mixed solution. PLGA was dissolved in 12 mL of dichloromethane to prepare 6.25% solution, followed by sonicated and emulsified at 12,000 rpm for 3 min. And then, the prepared NH_4_HCO_3_ solution was slowly dripped, and continued sonicating for 3 min. The primary emulsion was poured into 450 mL of 0.5% PVA solution and stirred overnight at 700 rpm for fully evaporation of organic solvent. The obtained microspheres were filtered with a strainer, washed with deionized water, and lyophilized for subsequent experiments.

### Preparation and characterization of hydrogel platform

Methylcellulose (gel) was dissolved in normal saline at a concentration of 14%, and stored at 4 ℃ until use. Then, porous microspheres (MPs) loaded hydrogel (Mgel) was prepared by mixing gel (14%) and MPs (4%). The hydrogel platform (IR820/Mgel) was prepared by mixing gel (14%), IR820 (100 μg/mL) and MPs (4%), respectively. The morphology of pre-gelled solution, gel, Mgel, and IR820/Mgel, as well as injectable ability of IR820/Mgel were obtained via digital camera. The surface and cross-sectional morphologies of the gel and Mgel were viewed using a scanning electron microscopy (SEM, JSM-5900LV, JEOL, Tokyo, Japan).

### Thermoresponsive and injectable behavior of hydrogel platform

Rheological measurements of the hydrogel platform were carried out with HAAKE MARS RS6000 rheometer (Thermo Scientific, Germany). The samples were placed on a circular testing table with a diameter of 40 mm, and the distance between the clamp and the shaft bottom platform was 1 mm. To investigate the thermoresponsive behavior of the variation in the storage (G’) and loss modulus (G’’) of gel and Mgel were examined as functions of temperature from 25 to 40 ℃ at a heating rate of 1 ℃/min, with a controlled stress of 4.0 dyn/cm^2^ and at a frequency of 1.0 Hz. In addition, gelation time of gel and Mgel at 37 ℃ was measured to detect their injectable ability. The gelation time was defined as the time when G’ became higher than G’’. The injectable ability of the hydrogel platform was also observed and recorded via digital camera.

### Photothermal properties of hydrogel platform

IR820/Mgels loaded with different concentrations (0 μg/mL, 50 μg/mL, 100 μg/mL and 150 μg/mL) of IR820 were irradiated using an 808 nm NIR laser (Laser Optoelectronics Technology Co., Ltd. Changchun, China) under 1 W/cm^2^ for 5 min. The temperature variation of IR820/Mgel was simultaneously detected using a Fluke Ti32 Infrared (IR) thermal camera system (Fluke, Everett, WA, USA).

### Live and dead staining assay

To evaluate the photothermal effects of the hydrogel platform, breast cancer cells (4T1) were seeded into a 24-well plate at an initial cell density of 1.0 × 10^5^ cells/mL for further incubated for 1 h. The following five different groups were designed: (1) control, (2) cells treated with hydrogel platform (IR820/gel), (3) cells treated with laser irradiation (L), (4) cells treated with free IR820 plus laser irradiation (IR820 + L), and (5) cells treated with hydrogel platform plus laser irradiation (IR820/gel + L). Among them, using the method of transwell, we added free IR820 or IR820/gel into the upper chamber. Then, put the chamber into the 24 well plate. After set aside for 10 min, groups with laser irradiation were irradiated using an 808 nm laser at a power density of 1 W/cm^2^ for 5 min. Then, survival and death of these treated cells were detected via a Live & Dead Viability/Cytotoxicity Assay Kit. Images were captured using a fluorescence microscope (Nikon Canada, Mississauga, Canada).

### In vitro cytotoxicity and in vivo biocompatibility of hydrogel platform

In vitro cytotoxicity of Mgel extract was examined by the MTT assay using two kinds of fibroblasts (L929 and NIH3T3 cells). Briefly, appropriate amount of culture medium was added to the upper layer of Mgel for hydrogel extraction. After 24 h, the extract liquid was collected and diluted with culture medium to different concentrations. L929 and NIH3T3 cells were cultured with different concentrations of Mgel extract liquid (0%, 25%, 50% and 100%, respectively) up to another 48 h. Then, the cell viability was determined by MTT method.

In vivo dorsal subcutaneous injection to BALB/c mice were used to investigate the biocompatibility of the gel and the MPs. The general conditions (activity, swelling, redness, and other clinical signs) and mortality of mice were carefully observed. There mice were sacrificed at different time points (3 d, 1 w, 2 w and 4 w, respectively). The shape and appearance of the gel at the injection site were observed and photographed. We also conducted subcutaneous injection of the Mgel and observed for 2 weeks. The tissues around the injection site were carefully removed, and fixed with 10% neutral formalin, followed by hematoxylin and eosin (H&E) staining for further histopathological examination.

### Detection the retention of IR820 loaded hydrogel platform

Fluorescence images were captured by an IVIS Lumina III imaging system (Perkin Elmer, Caliper Life Sciences, MA, USA) to detect the retention of IR820 loaded hydrogel platform. The mice were randomly divided into two groups (six mice per group), and injected with 100 μL IR820 solution or IR820/gel. The concentration of IR820 and gel was 100 μg/mL and 14%, respectively. Mice of each group were anesthetized, and fluorescence images were obtained with maximal excitation and emission wavelengths at 710 nm and 820 nm, respectively at the determined time points (1 h, 2 h, 4 h, 6 h, d2, d3, d4, d5, d6, d7). All the images were analyzed using onboard software.

### Surgical procedure and in vivo photothermal therapy

4T1 cells (1 × 10^6^ cells per mouse) were injected into the right mammary fat pads of mice to build orthotopic primary breast tumors. When the volume of tumors reached about 300 mm^3^, tumors were partially debulked for producing post-surgical residual tumor model according to the previous studies [39, 40]. Briefly, mice were anesthetized, with the surgical area sprayed with povidone iodine. And then, approximately 90% of the tumor was removed, leaving 10% residual tissue behind. Subsequently, mice were randomly divided into five groups: (G1) Control, (G2) IR820/Mgel, (G3) L, (G4) Free IR820 + L, (G5) IR820/Mgel + L. The hydrogel platform was injected directly beside the residual tumor. Mice in G3 to G5 were irradiated by the 808 nm NIR laser at 1 W/cm^2^ for 5 min. During the irradiation process, the temperature of injection site was recorded by an IR thermal camera. Tumor volume and body weight of all mice were recorded every other day. The tumor volume was calculated using the following formula: tumor volume (mm^3^) = length × (width)^2^ × 0.5. Fourteen days after treatment, tumors and major organs (heart, liver, spleen, lung, and kidney) of mice in different groups were collected and fixed for further H&E staining. The tumor tissues were investigated with Ki67 staining. In addition, complete blood counts and blood biochemistry analysis were performed to investigate the potential side-effects.

In order to verify the phototoxicity of the IR820/gel to skin and normal tissues around the tumor, eight of BALB/c mice were inoculated subcutaneously with 4T1 cells, and were randomly divided into the control group and the experiment group. The tumor-bearing mice went through the surgical procedure that described above. Among them, mice of the experiment group were injected with IR820/gel into tumor bed and then irradiated by the 808 nm NIR laser at 1 W/cm^2^ for 5 min. The irradiation area was continuously observed to evaluate the condition of the local skin. At the end, the surrounding skin and subcutaneous tissue were collected for H&E staining to observe the degree of tissue inflammation and whether there were pathological changes.

### In vitro cell attachment property and in vivo filling effect of hydrogel platform

Normal breast epithelial cells (MCF-10A) and adipose stem cells were co-cultured with MPs to investigate the ability of the hydrogel platform as the cell anchorage. In brief, 1 mg of MPs were co-cultured with MCF-10A cells or adipose stem cells in a 96-well plate at a cell density of 1 × 10^5^ cells/mL. After 24 h, MPs with cells were fixed in 2.5% glutaraldehyde for 30 min and dehydrated by freeze-drying to observe the morphology of cells attached MPs via SEM.

Nine female BALB/c mice were employed to evaluate the in vivo filling effect of the hydrogel platform as a breast filler. Gel at a volume of 200 μL was administered by dorsal subcutaneous injection. Three mice were sacrificed at 1 w, 2 w and 4 w, respectively, and the injection site were opened with a surgical scissors to observe and photograph the state of gel.

### Statistical analysis

The statistical analysis was performed by one-way analysis of variance (ANOVA) for multiple group comparisons using SPSS 20.0 software (Chicago, IL, USA). Data were presented expressed as mean ± standard deviation (Mean ± SD), wherein significance is indicated by *P* value < 0.05 (*), *P* < 0.01 (**), and *P* < 0.001 (***). *P* < 0.05 was considered to indicate statistical significance.

## Results and discussion

### Preparation and characterization of MPs and hydrogel platform

**Fig. 1:**
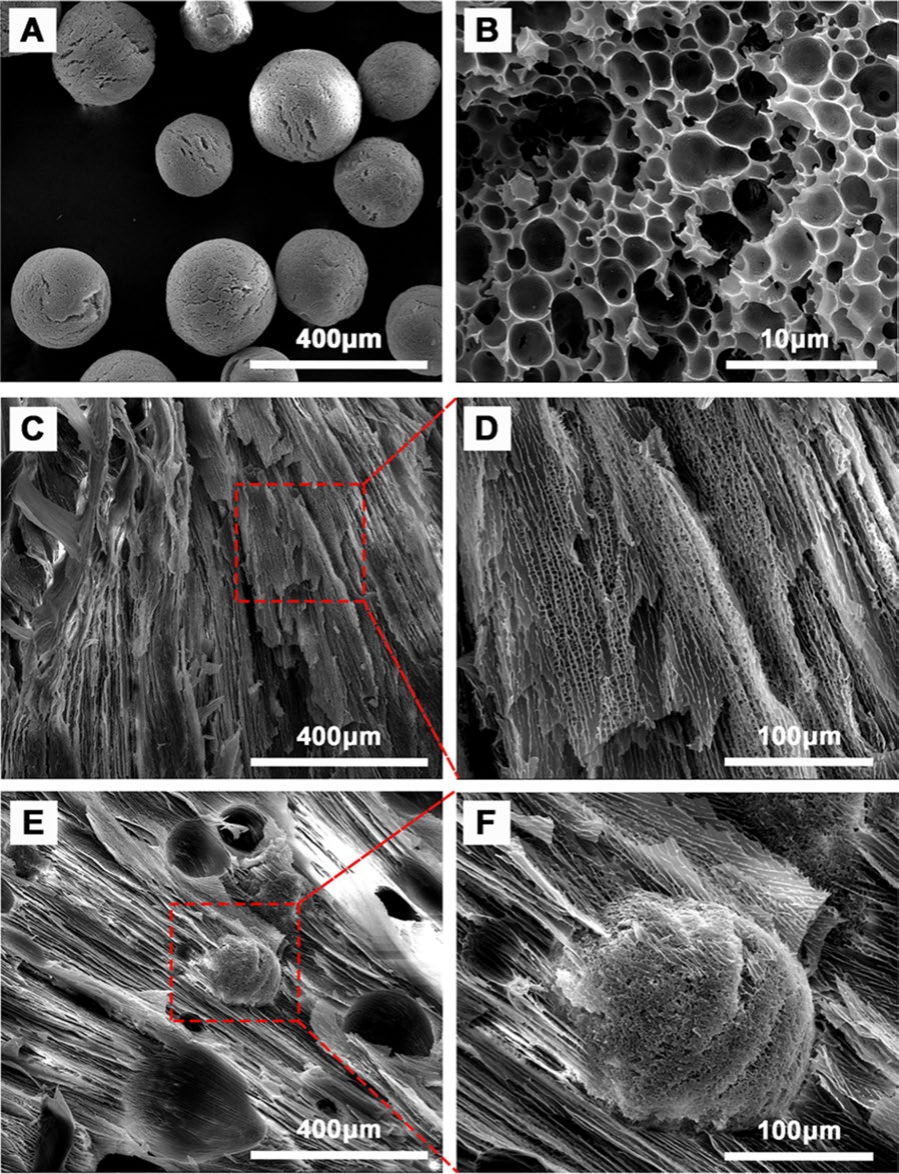
SEM micrographs of PLGA MPs (**A**, **B**), gel (**C**, **D**), and Mgel (**E**, **F**)

First, MPs were synthesized by a single emulsion/evaporation method. From the SEM images in Fig. 1A, the morphology of MPs possessed round shape with diameter 208 ± 37 μm. The inter-structure of MPs was porous, which could be observed from Fig. 1B. The average pore size in MPs was about 2.8 μm. The porous MPs were translucent, which was characterized via optical microscope (Additional file 1: Figure S1A).

The micro-architecture of gel and Mgel were examined via SEM. All the samples were verified to have an interconnected porous network structure (Fig. 1C–F), which is vital for the efficient loading of small molecule compound. The SEM confirmed that MPs were distributed in hydrogel samples (Fig. 1E and F). IR820/Mgel were developed by simply mixture of gel, MPs and IR820. The morphology of gel, Mgel and IR820/Mgel were characterized by photograph. As shown in Additional file 1: Figure S1B, when the temperature was lower than the gelation temperature, pre-gelled solution of the gel was free-flowing. When laid at 37 ℃ for half an hour, the hydrogel platform quickly transformed into a non-flowable gel-like state whether or not MPs and (or) IR820 were added in. It suggested that MPs and IR820 did not affect the crosslinking of gel.

### The rheological properties of hydrogel platform

**Fig. 2:**
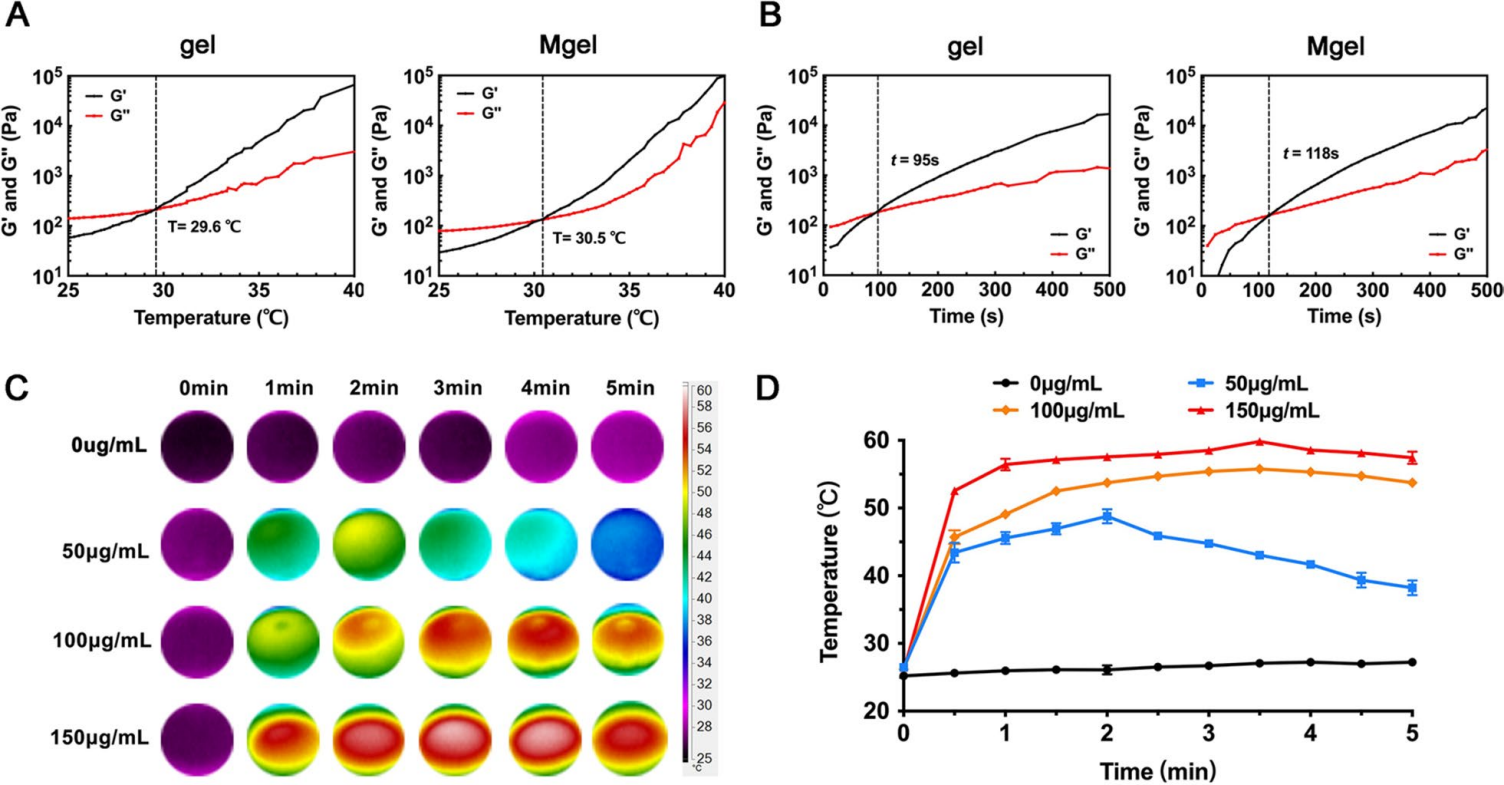
The rheological properties and in vitro photothermal properties of hydrogel platform. **A** The thermoresponsive behavior of gel and Mgel. **B** The sol–gel phase transition time of gel and Mgel. **C** IR thermographic images of Mgel with different concentrations of IR820 after exposure to an 808 nm laser at a power density of 1 W/cm^2^. **D** The temperature–time profile of IR820/Mgel with different concentrations of IR820

The rheological behavior of gel and Mgel were further characterized. Firstly, to investigate the thermoresponsive behavior of the hydrogel platform, the variation in the storage (G’) and loss modulus (G’’*)* of gel and Mgel were examined during the gelation process over a temperature range from 25 to 40 ℃. As shown in Fig. 2A, both the G’ and G’’ values of gel and Mgel increase rapidly, and the increase rate of G’ was greater than that of G’’, indicating that the hydrogel platform began to undergo a sol–gel phase transition. In particular, the intersection points between the two temperature curves implied the occurrence of sol–gel transition. It meant the loading of MPs did not influence the sol–gel phase transition of gel. The sol–gel transition temperatures of gel and Mgel were analyzed to be 29.6 ℃ and 30.5 ℃, respectively. With the temperature rising, G’ and G’’ continued to increase, reflecting that the strength of the hydrogel platform gradually increased. Compared to gel, Mgel went through a similar change in sol–gel transition temperatures, while Mgel exhibited a slightly increase in G’ and G’’ partly due to the loading of MPs. It meant the porous MPs could help to increase the gel strength and also provided indirect evidence for MPs to interact with the gel. Thus, the temperature of sol–gel phase transition and the gelation intensity could allow for stabilization of the breast filler for breast reconstruction.

Gelation time measurement was also examined and implied from Fig. 2B that gel could accomplish its sol–gel phase transition within 95 s at 37 ℃, while gelation time was 118 s for Mgel. In addition, we provided an image showing the injectability of the pre-gelled solution of hydrogel platform through a syringe (Additional file 1: Figure S1C). These changes of rheological properties of Mgel did not affect the application in cancer therapy. Firstly, the loading of MPs did not influence the sol–gel phase transition of gel. Secondly, MPs could help to increase the strength of gel to realize a long-term in situ function in vivo. Thirdly, MPs were explored as the anchor points for normal breast epithelial cells and adipose stem cells, which may provide benefits for tissue reconstruction. Therefore, appropriate sol–gel phase transition temperature and time of Mgel were favorable for its application as an injectable in situ hydrogel in vivo.

### In vitro photothermal properties of hydrogel platform

The photothermal effect of the hydrogel platform that prepared with different concentrations of IR820 was investigated through detecting the trend of the temperature variation under the irradiation of an NIR laser (808 nm). The data indicated that the hydrogel platform containing IR820 exhibited excellent photothermal effect. As indicated in Fig. 2C and D, the temperature of IR820/Mgel with 100 μg/mL of IR820 under the irradiation of a 1 W/cm^2^ laser rapidly increased, reaching 49.07 ± 0.29 ℃ at 1 min. Then, temperature was relatively stable and remained above 50 ℃, with the highest temperature at 55.77 ± 0.38 ℃. After 5 min of irradiation, the temperature of IR820/Mgel was 53.73 ± 0.35 ℃. When the concentration of IR820 was 150 μg/mL, the temperature of the platform rapidly increased from 25.97 ± 0.23 ℃ to 56.43 ± 0.84 ℃ during the first minute, and remained above 57 ℃ after then. During irradiation, the highest temperature was 59.83 ± 0.15 ℃. However, the highest temperature of IR820/Mgel with 50 μg/mL of IR820 was 48.80 ± 1.04 ℃ during irradiation, and the temperature gradually decreased to 38.23 ± 1.10 ℃ at the fifth minute. This may be related to the photobleaching of IR820, that is, photothermal effect of the photosensitizer decreased with the prolongation of irradiation time. To some extent, probably because the increase rate of the temperature was slower than the heat diffusion rate when the concentration of photosensitizer was low. By comparison, the temperature of pure Mgel was almost unchanged, fluctuating between 25.20 ± 0.36 ℃ and 27.23 ± 0.31 ℃. In fact, too low temperature was not enough to cause damage to tumor cells, while too high temperature may damage normal tissues around tumors. From these data, it could be seen that IR820/Mgel with 100 μg/mL of IR820 had a relatively suitable hyperthemia after irradiation.

**Fig. 3:**
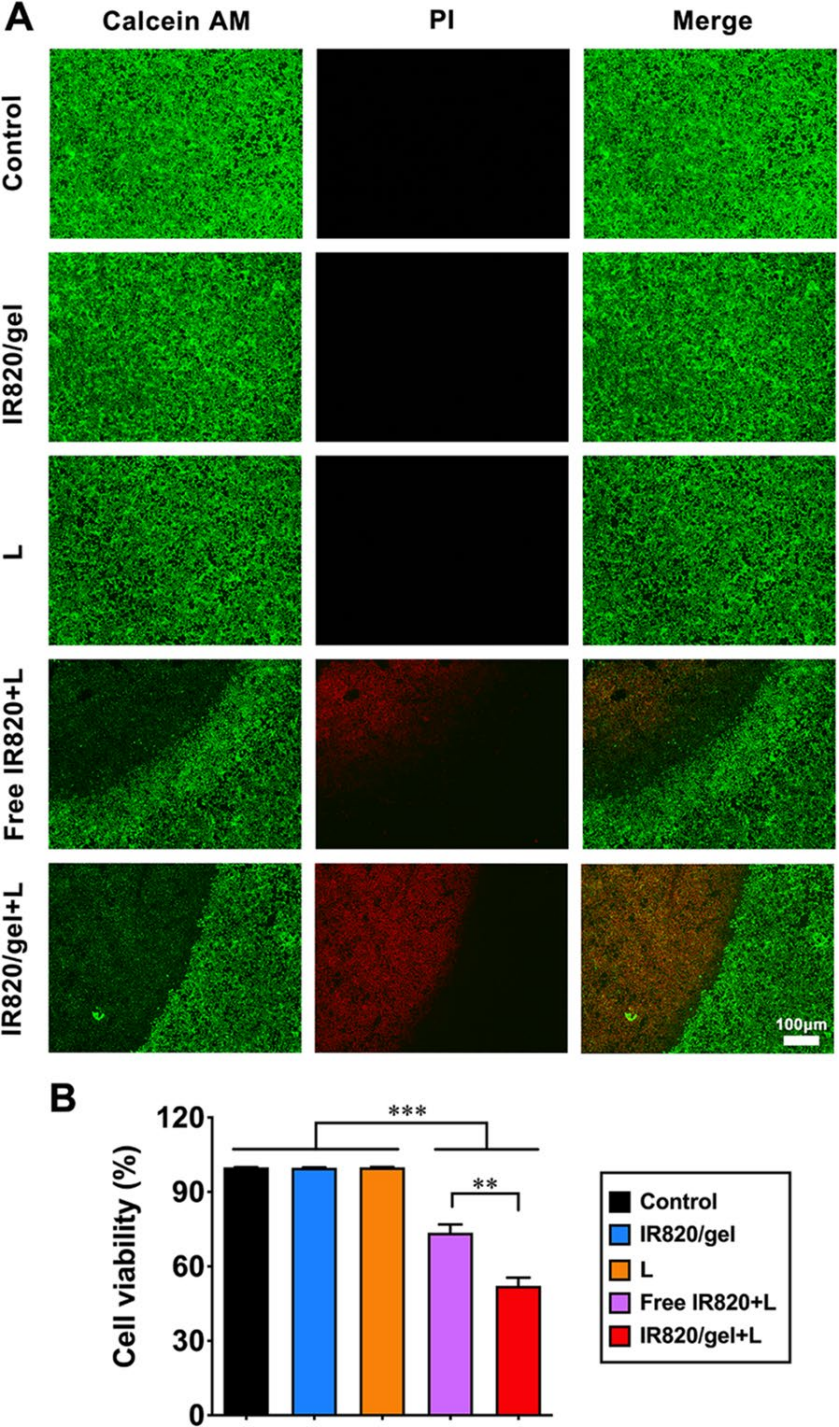
In vitro photothermal effect of hydrogel platform. **A** The images of live and dead 4T1 cells stained by calcein-AM/PI (green signal, live cells; red signal, dead cells) with various treatments. The concentration of IR820 was 100 µg/mL, and the laser power density was 1 W/cm^2^ (808 nm, 5 min). **B** Calculated liveratios of 4T1 cells with various treatments. *P* < 0.01 (**), and *P* < 0.001 (***)

To evaluate the photothermal effect of the hydrogel platform on the viability of breast cancer cells (4T1), calcein-AM and PI were used to stain live and dead cells. As shown in Fig. 3A, 4T1 cells of control, IR820/gel or laser group, presented intense green fluorescence, implying high cell viability. By comparison, IR820+L could also produce photothermal effect and kill tumor cells. But IR820/gel+L presented more powerful killing effect with a significantly lower survival rate than those of any other groups (Fig. 3B). It may because that free IR820 was released and diffused more quickly from the upper chamber to the lower chamber, and the concentration of IR820 in the laser area decreased, which lead to a reduction of the photothermal effect. Therefore, the result of live and dead staining assay not only revealed that photothermal performance of the hydrogel platform, but also suggested that the hydrogel platform helped to prolong retention time of IR820 and played a positive role of maintaining the concentration of photosensitizers at a high level in local area.

**Fig. 4:**
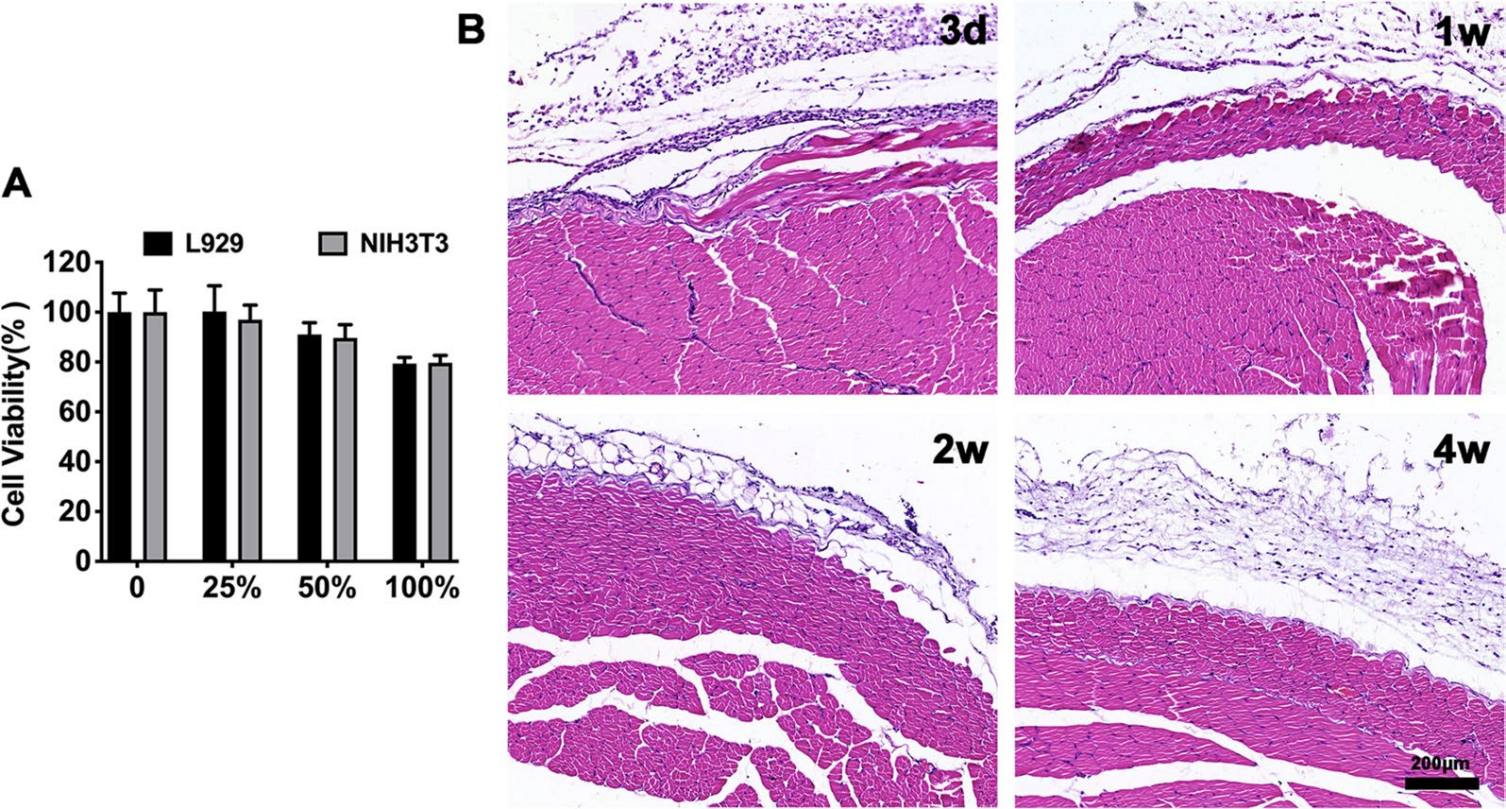
In vitro and in vivo biocompatibility of hydrogel platform. **A** Effect of hydrogel extracts on cell viability measured by MTT assay. **B** Histological observation of biocompatibility assay at different time points

### In vitro and in vivo biocompatibility of hydrogel platform

The biocompatibility of Mgel in vitro was investigated using two types of fibroblasts (L929 and NIH3T3 cells) via MTT method. As presented in Fig. 4A, with the concentration of Mgel extracts increased, the cell survival rate decreased. However, even under the highest dose, the viability of L929 and NIH3T3 cells were 79.35% and 79.68%, respectively. The results further demonstrated that the hydrogel platform had no obvious toxicity for fibroblasts.

In addition, dorsal subcutaneous injection to BALB/c mice were conducted for examining the biocompatibility in vivo of the gel. During the observation period, there was no swelling or redness around the injection area by gross observation. As presented in Fig. 4B, the results of histopathology examination showed that there was only mild infiltration of inflammatory cells and no obvious pathological changes surrounding the injection site, which indicated that the inflammatory reaction of the gel was slight and it had excellent biocompatibility.

As for the MPs, numerous studies have reported that the degradation of the polymer MPs was very slowly, which could long-term retention in the local tissue with a good biocompatibility [22, 41, 42]. In our previous study, the biocompatibility and biodegradation of the microsphere/IR820 hybrid hydrogels have been investigated, the results also showed that it has a good biocompatibility, the inflammation gradually subsided later in the process and the biodegradation process was slow [23]. In this study, the biocompatibility of MPs from the body was also evaluated. As shown in Additional file 2: Figure S2, we observed that there were a large number of inflammatory cells accumulated around the materials, which were mainly neutrophils, indicating an acute inflammatory response of the organism. This was due to the mechanical damage of the syringe and the implantation of the materials, which was a normal reaction of the body. Meanwhile, we performed Masson staining of the skin at the injection site and found that the material stimulated the production of fibers after implantation under the subcutaneous skin (Additional file 2: Figure S2). At the same time, the MPs could play a role in padding the breast, and their degradation and slow metabolism could help in breast reconstruction.

### The retention effect of IR820 loaded hydrogel platform

**Fig. 5:**
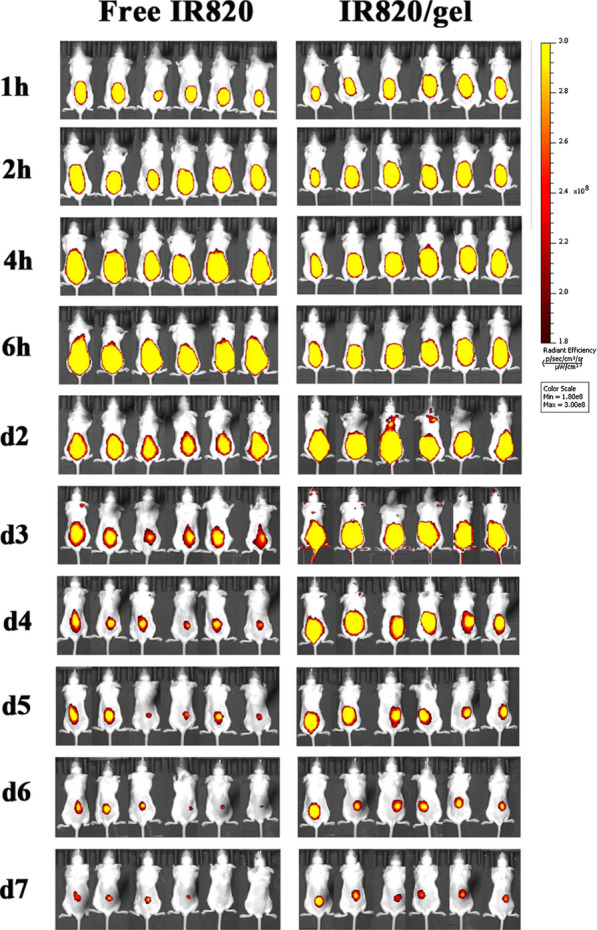
The retention effect of hydrogel platform. In vivo distribution and retention profile of IR820/gel via intravital fluorescence imaging

A hydrogel platform could act as a reservoir and retain the encapsulated agents at the injection sites, thereby help to maintain the sufficient therapeutic concentration of them. Therefore, we investigated the in vivo distribution and retention profile of IR820/gel after subcutaneous injection via intravital fluorescence imaging. As shown in Fig. 5, the fluorescence areas of free IR820 gradually increased from the 1st to the 6th hour due to its liquidity, while the fluorescence area of IR820/gel group did not change much due to its quickly transformed into a semi-solid state after subcutaneous injection. The fluorescence intensity of both groups declined gradually over time from d2 to d7, and free IR820 group decreased obviously at d2. While it was not until d4, the fluorescence intensity of IR820/gel group decayed significantly. It suggested that the hydrogel platform displayed prolonged retention in subcutaneous tissue, which was essential for the PTT. On one side, the prolonged retention time of the gel could maximize the therapeutic effect by maintaining the concentration of photosensitizers at a high level in the tumor resection bed. On the other side, it could minimize the potential off-target toxicity caused by distribution of photosensitizers in the neighboring tissues. Thus, the results revealed that the hydrogel platform could be utilized as a favorable carrier of IR820, which made it possible to be used for the PTT.

### In vivo photothermal performance and anti-tumor efficiency of hydrogel platform

**Fig. 6:**
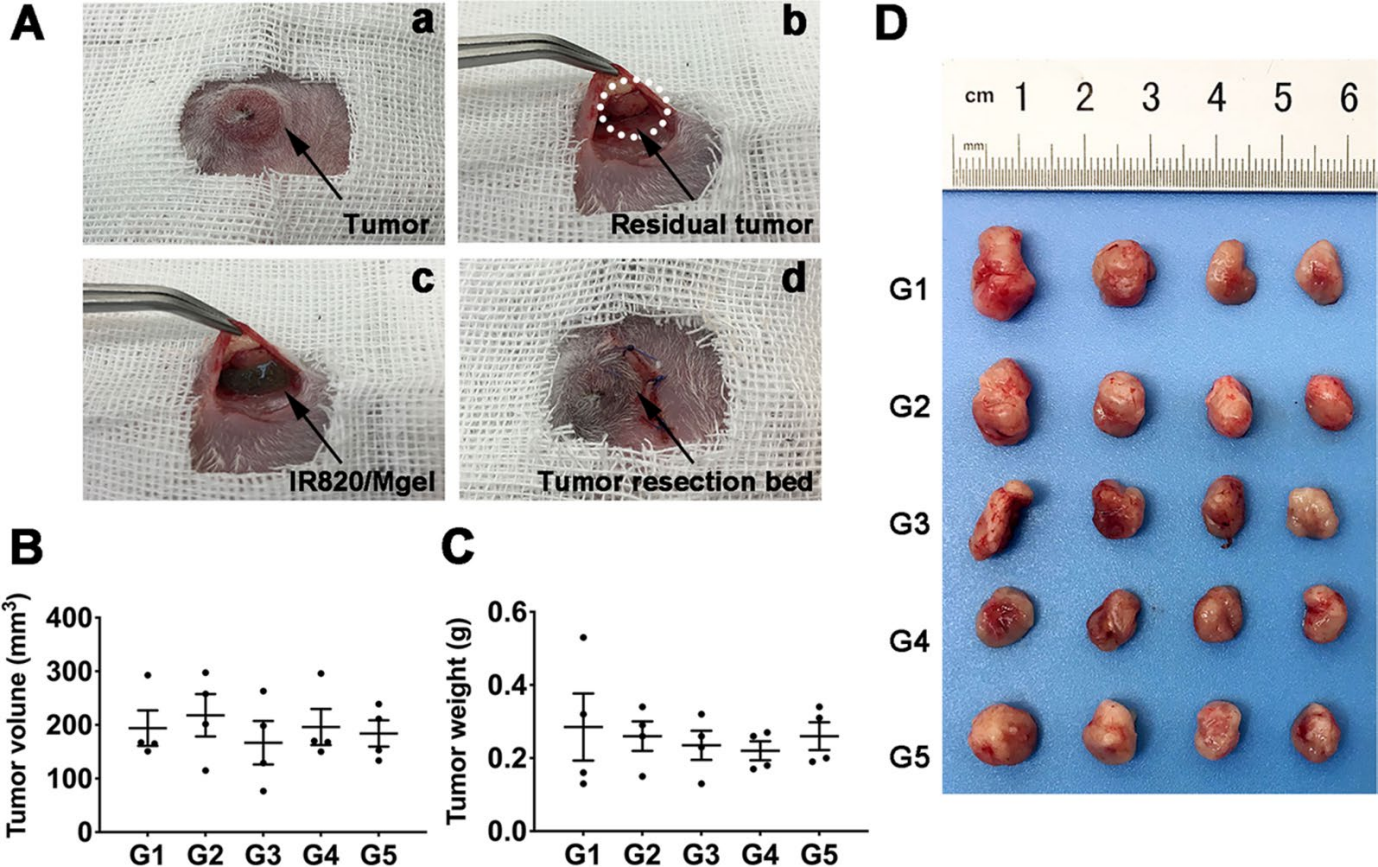
The surgical procedure. **A** Tumor resection and hydrogel platform injection procedure. **a** Surgery was performed; **b** Mimicked incomplete tumor removal; **c** The hydrogel platform was injected into the tumor cavity;**d** The wound was closed. Strategies to control resected tumor size: **B** Tumor volume was accurately recorded before surgery, and mice with similar tumor volume were selected for randomly grouping; **C** A single experimenter performed the surgery to remove about 90% of the tumor volume; **D** Resected tumors were weighed and photographed to ensure the size of the residual tumors in each group are relatively uniform

**Fig. 7:**
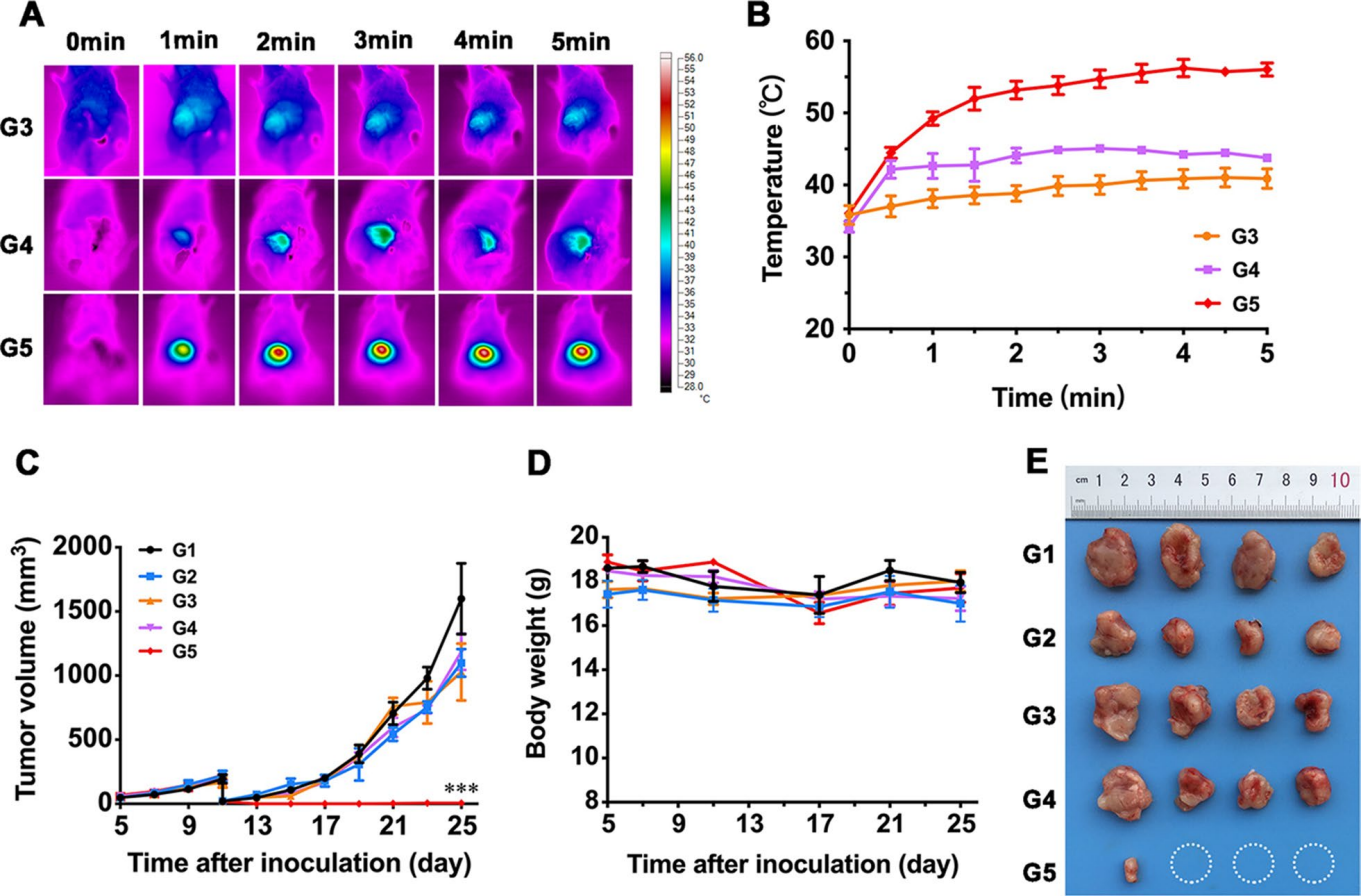
In vivo photothermal activities in a tumor-bearing model and anti-tumor efficacy of the hydrogel platform mediated photothermal therapy. **A** The dynamic photothermal images and **B** temperature–time profile at the tumor site under NIR irradiation (808 nm, 1 W/cm^2^, 5 min). **C** Tumor growth curves and **D** body weight of mice following various treatments. **E** Excised tumors image at day 14 after surgery and administration. G1, Control; G2, IR820/Mgel; G3, L; G4, Free IR820+L; G5, IR820/Mgel+L. *P* value < 0.001 (***)

The anti-tumor efficiency of the hydrogel platform on breast cancer was further investigated in vivo. In clinical practice, it is inevitable to face the situation that some of the tumor could not be removed completely by surgery, especially for advanced-stage cancers. We simulated the condition by performing the surgery in the manner shown in Fig. 6A. In addition, we excluded the quality control system according to the previous studies and adopted the method shown in the following Fig. 6B–D in order to ensure the relative homogeneity of the surgical procedure and the postoperative tumor volume. Approximate 90% of the primary tumor was removed, and the hydrogel platform was injected into the dissected empty space. In this procedure, mice with similar tumor volumes were selected for random grouping. A single experimenter performed the surgery to remove about 90% of the tumor volume, with the same size residual tumors for further treatment. After surgical intervention, IR820 loaded hydrogel platform was filled in the tumor resection bed and received NIR irradiation with an 808 nm laser at 1 W/cm^2^ for 5 min. As indicated in Fig. 7A and B, the temperature of IR820/Mgel+L group quickly reached 53.2 ℃ within first 2 min, and then remained around 53.0 ℃ during the procedure. However, the temperature of free IR820+L group remained about 44.0 ℃ during 5 min. Therefore, the hydrogel platform showed a good in vivo photothermal stability.

After 14 days continuous observation, the outcomes of different treatments were analyzed. The recurrent breast tumor volume in G5 was significantly inhibited (Fig. 7C), which was consistent with the tumor photograph (Fig. 7E). Especially, the tumor volume in G5 was significantly smaller than that in G4. In addition, Fig. 7E also displayed that tumor of three mice disappeared after treatment of IR820/Mgel hydrogel platform plus irradiation. The recurrent tumor suppression rate of G5 reached as high as 75%. Thus, these results suggested that the hydrogel platform provided a practical paradigm for preventing breast tumor recurrence after surgery.

**Fig. 8:**
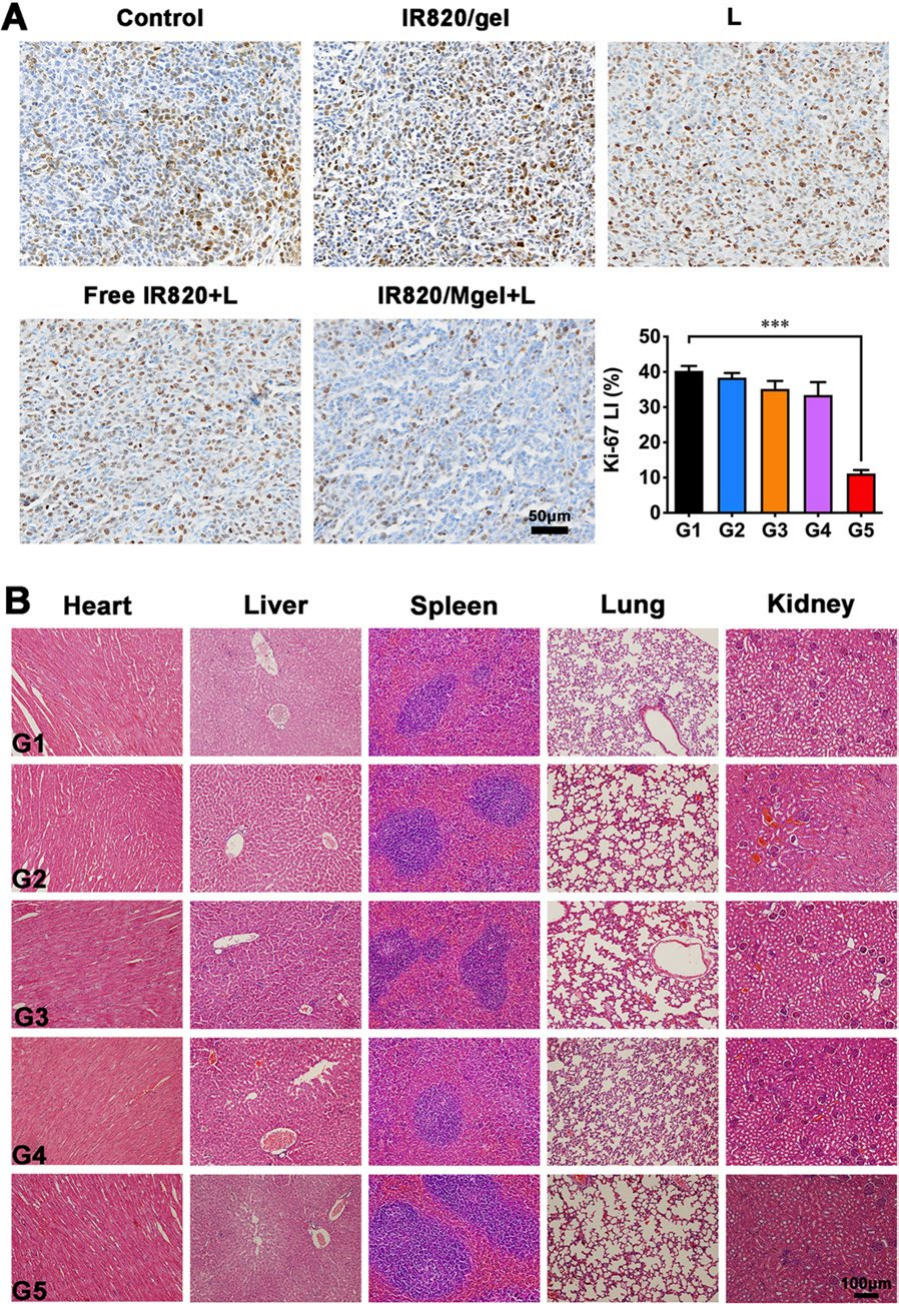
Histological analysis of the tumor tissues and major organs. **A** Ki67 immunohistochemical staining and representative mean Ki67 LI in each group; **B** H&E staining of major organs of tumor-bearing mice receiving different treatments. G1, Control; G2, IR820/Mgel; G3, L; G4, Free IR820+L; G5, IR820/Mgel+L. *P* value < 0.001 (***)

Immunohistochemical Ki67 staining of the tumor sections was used to evaluate the tumor cell proliferation (Fig. 8A). Visually and statistically, tumor cells in G5 exhibited much lower Ki67 expression, which was presented with Ki67 labeling index (Ki67 LI), indicating markedly reduced tumor cell proliferation after treatment. Additionally, the biosafety of the hydrogel platform was investigated. The mice showed no abnormal behaviors and noticeable weight loss (Fig. 7D). From H&E staining results, there was no visible damage observed in the major organs that collected from all groups after treatments (Fig. 8B). In addition, Additional file 3: Figure S3, Additional file 4: Figure S4 indicated that there was no significant difference in complete blood counts and blood biochemistry analysis results among all groups, suggesting no obvious systemic toxicity of the hydrogel platform.

In order to verify whether there was phototoxicity of IR820/gel to skin and normal tissues around tumor, we took pictures daily of the mice which went through surgical procedure and irradiation to observe the incision healing, and collected the surrounding tissues for H&E staining to estimate the pathological changes. As shown in Additional file 5: Figure S5, the skin of the incision area did not show obvious redness and ulceration during a seven-day observation period after irradiation, and the incision healed well. Meanwhile, we could notice that the tumors in control group grew more rapidly compared with experimental group, indicating that the experimental group had a well local control rate. After that, the mice were sacrificed and the skin at the incision area were collected for H&E staining (Additional file 6: Figure S6). We found that there was only mild infiltration of inflammatory cells and no obvious pathological changes in surrounding tissue of the irradiation site of IR820/gel groups, suggesting that there was no serious phototoxicity in local tissues. In addition, considering the adjacent relationships of organs, we furtherly assessed whether the irradiation had a certain effect on lungs. The H&E staining showed that there was no difference in the histology of lung tissues between two groups.

To sum up, in vivo anti-recurrent experiment demonstrated a novel strategy based on a photothermal hydrogel platform could function as a promising treatment for preventing locally recurrence of breast cancer, and had good tolerance and biological safety in mice.

### In vitro and in vivo breast construction property of hydrogel platform

**Fig. 9:**
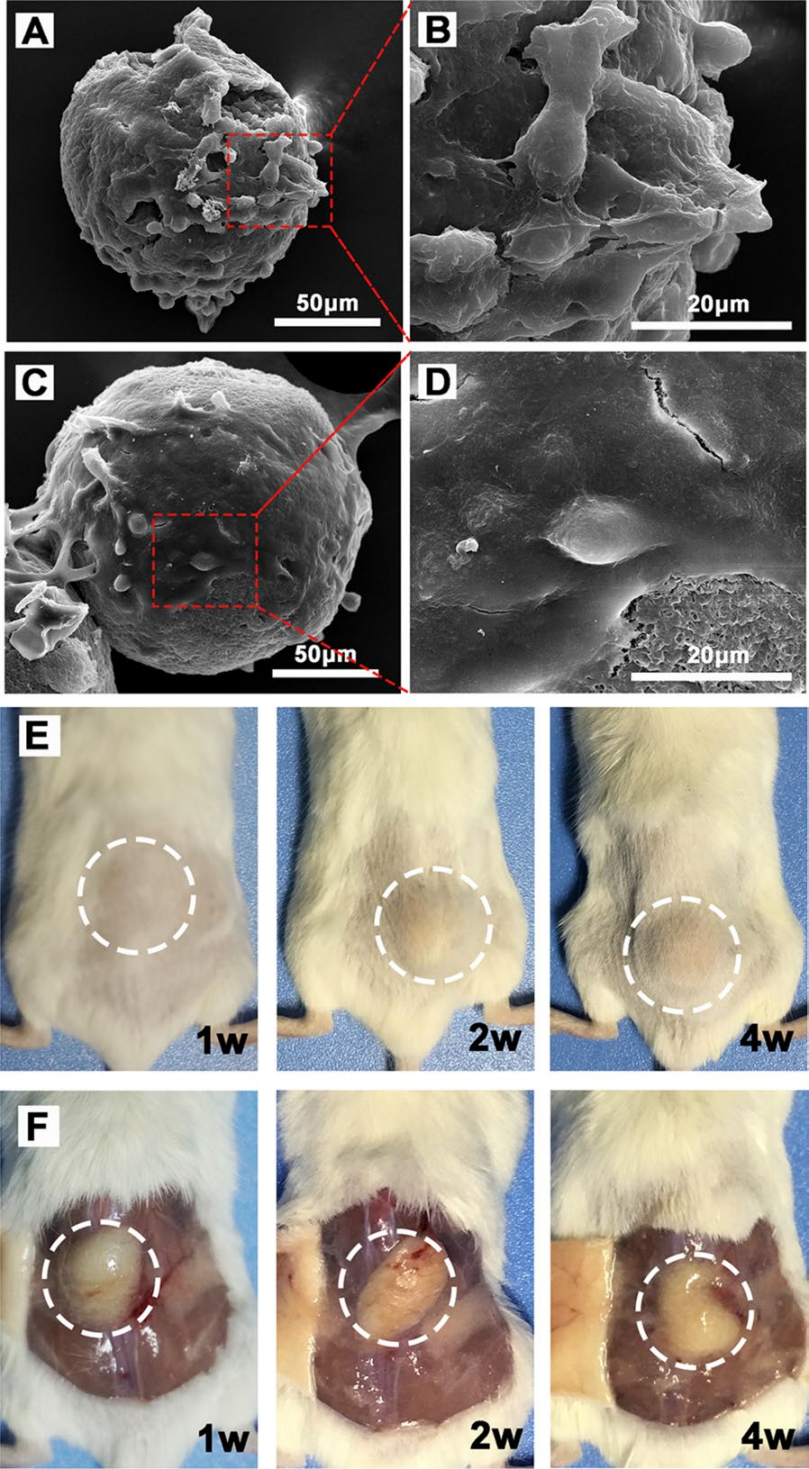
In vitro and in vivo breast construction property of hydrogel platform. **A**–**D** SEM micrographs of MCF-10A cells adhered MPs. **E**, **F** In vivo shaping and morphology change of the gel

As a potential breast reconstruction material, the hydrogel platform should be a favorable filler for dissected empty space after surgery, and had the capability to support the attachment of normal breast cells and adipose cells for the following tissue repair. Therefore, the ability of MPs as cell anchor points that showed improvement in cell adhesion was further investigated. The MCF-10A and adipose stem cells were seeded on plates and co-cultured with MPs for 24 h. And then, cell attachment on MPs was assessed by the SEM. According to the results, MPs were able to support MCF-10A cells (Fig. 9A–D) and adipose stem cells (Additional file 7: Figure S7) to attach, at the same time, these cells became a bridge connecting MPs, which formed a good scaffold for tissue reconstruction.

In addition, well shaping in vivo and slow degradation performance could also provide support for breast reconstruction. The in vivo shaping and filling effect of hydrogel platform were investigated by dorsal subcutaneous injection. On gross observation form Fig. 9E and F, the pre-gelled solution of the hydrogel platform quickly transformed into a gel state, forming a round-like gel mass under the skin after subcutaneous injection, and not spread around the injection site. For up to 4 weeks, there was no significant change in the morphology of the gel, which laid the foundation for prolonging the local retention of porous MPs and providing a long-term and stable cell adhesion scaffold. Therefore, these preliminary works indicated the potential ability of the hydrogel platform to be as a filler and cell anchorage for breast reconstruction.

## Conclusion

In summary, we have successfully developed a thermoresponsive and injectable hybrid hydrogel platform by incorporating porous MPs on the basis of the IR820 loaded methylcellulose hydrogel for anti-cancer PTT and breast reconstruction. With the presence of biocompatibility PTT agent, IR820/Mgel could quickly heated under NIR irradiation, enabling killing effect on 4T1 cells in vitro and prevention on post-surgical tumor recurrence in vivo. Meanwhile, the filling effect of the hydrogel platform could have a shaping effect on the residual cavity after breast surgery, and the contained porous MPs provide long-term support for cell adhesion to facilitate breast reconstruction. Therefore, the hybrid hydrogel platform offers a viable postoperative PTT strategy for the treatment of local recurrence of breast cancer, and also an alternative candidate strategy for postoperative care in women.

## Supplementary Information

**Additional file 1: Figure S1.** Characterization of porous MPs and hydrogel platform. (A) Morphological characterization of the porous MPs by optical microscope. (B) Sol–gel phase transition of the hydrogel platform. a: solution phase of gel; b: gel phase of gel; c: gel phase of Mgel; d: gel phase of IR820/Mgel. (C) Injectability of the hydrogel platform.

**Additional file 2: Figure S2.** Tissue reactions to Mgel by subcutaneous injection after 1w and 2w. Black arrow, MPs; Blue arrow, inflammatory cells; Yellow arrow, fibers.

**Additional file 3: Figure S3.** Complete blood counts of each group. (A) White blood cell (WBC) count; (B) Red blood cell (RBC) count; (C) Hemoglobin (HGB) concentration; (D) Platelet (PLT) count.

**Additional file 4: Figure S4.** Blood chemistry analysis of each group. (A) Alanine aminotransferase (ALT); (B) aAspartate aminotransferase (AST); (C) Creatinine (CRE); (D) UREA.

**Additional file 5: Figure S5.** Phototoxicity of IR820/gel to skin and normal tissues around tumor.

**Additional file 6: Figure S6.** H&E staining of tissues of incision area and lungs.

**Additional file 7: Figure S7.** In vitro breast construction property of hydrogel platform. The SEM micrographs of adipose stem cells adhered porous MPs.

## Data Availability

All data generated and analyzed during this research and included in this published article.
