# Supplementary figures and images for: Photothermal hydrogel platform for prevention of post-surgical tumor recurrence and improving breast reconstruction
Source: J Nanobiotechnology. 2021 Oct 7;19:307. doi: 10.1186/s12951-021-01041-w (PMC8499550; doi:10.1186/s12951-021-01041-w)

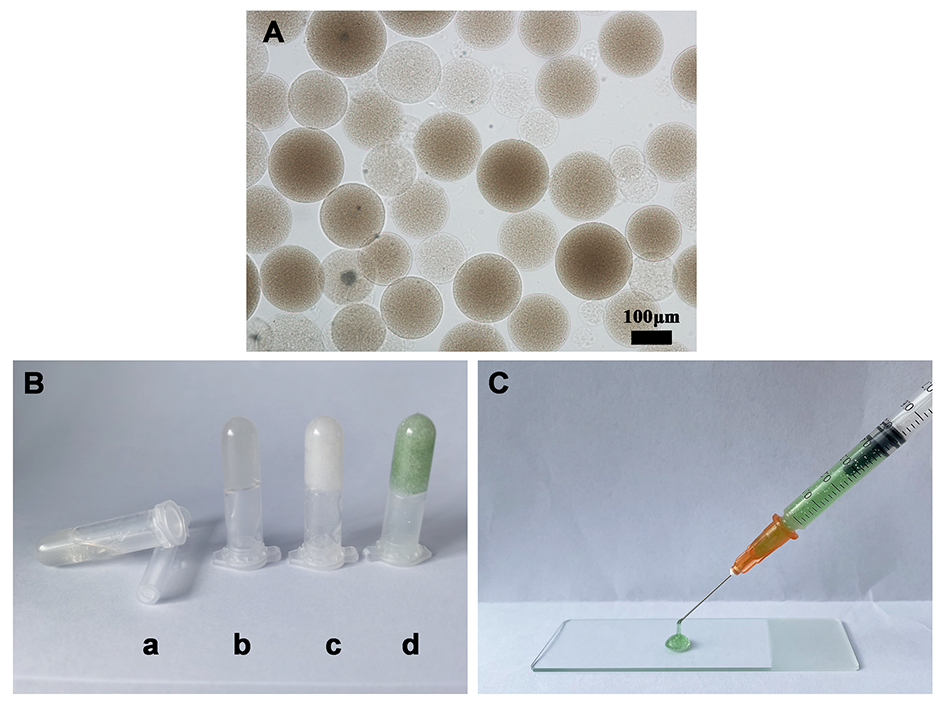

Supplement: Supplementary file 1 — Additional file 1: Figure S1. Characterization of porous MPs and hydrogel platform. (A) Morphological characterization of the porous MPs by optical microscope. (B) Sol–gel phase transition of the hydrogel platform. a: solution phase of gel; b: gel phase of gel; c: gel phase of Mgel; d: gel phase of IR820/Mgel. (C) Injectability of the hydrogel platform. [file 12951_2021_1041_MOESM1_ESM.tif]

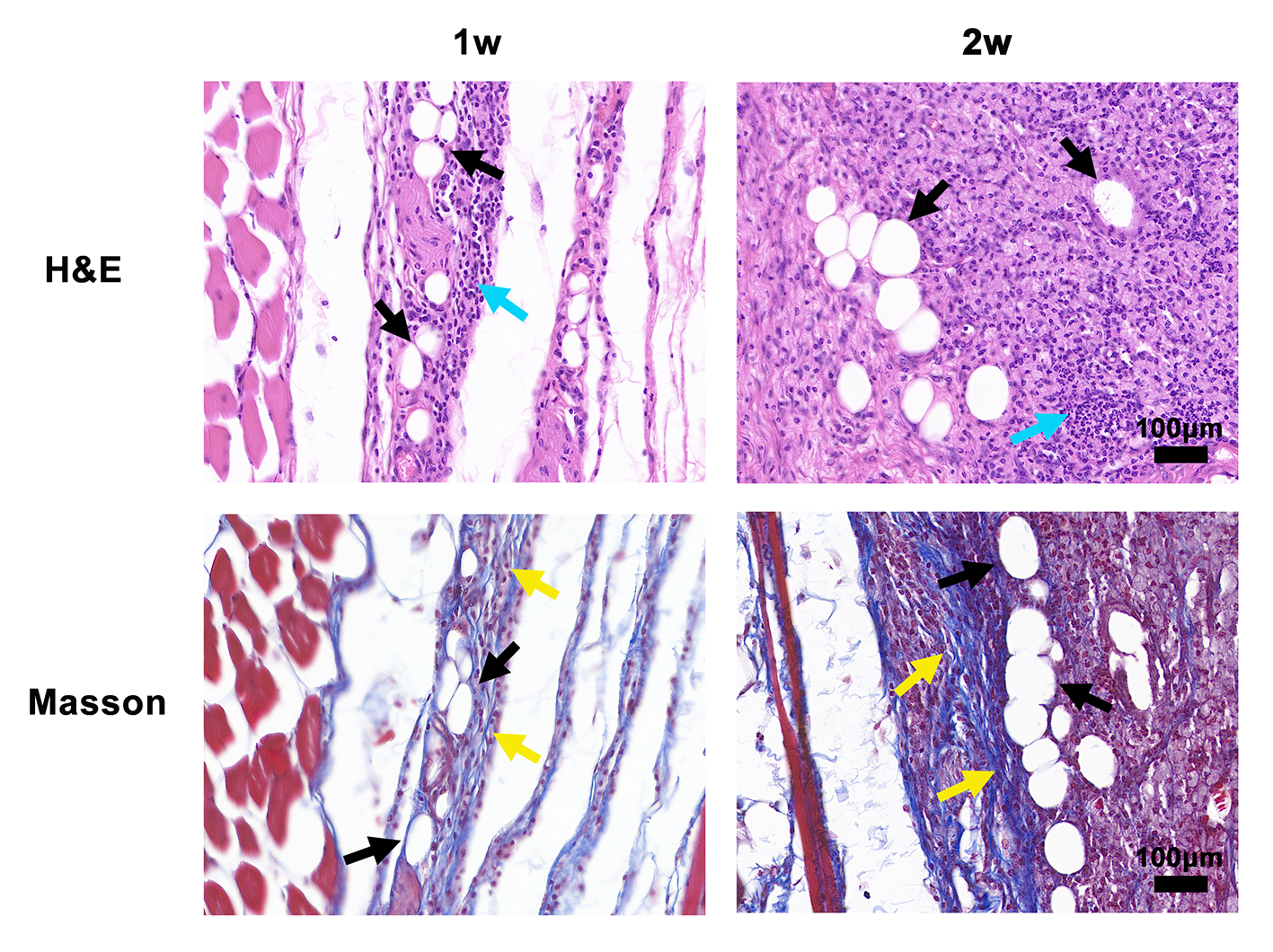

Supplement: Supplementary file 2 — Additional file 2: Figure S2. Tissue reactions to Mgel by subcutaneous injection after 1w and 2w. Black arrow, MPs; Blue arrow, inflammatory cells; Yellow arrow, fibers. [file 12951_2021_1041_MOESM2_ESM.tif]

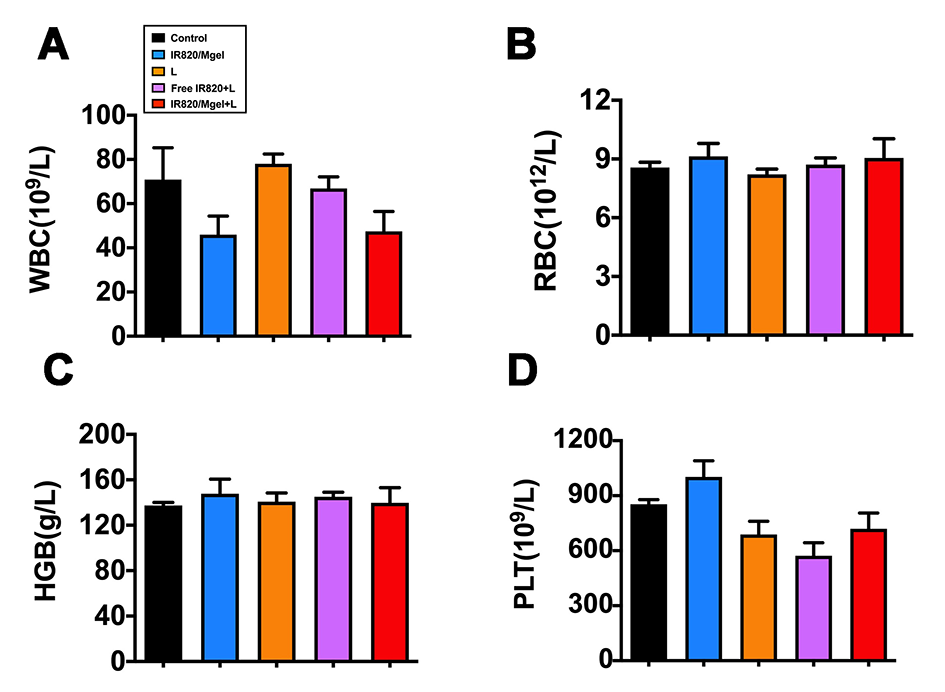

Supplement: Supplementary file 3 — Additional file 3: Figure S3. Complete blood counts of each group. (A) White blood cell (WBC) count; (B) Red blood cell (RBC) count; (C) Hemoglobin (HGB) concentration; (D) Platelet (PLT) count. [file 12951_2021_1041_MOESM3_ESM.tif]

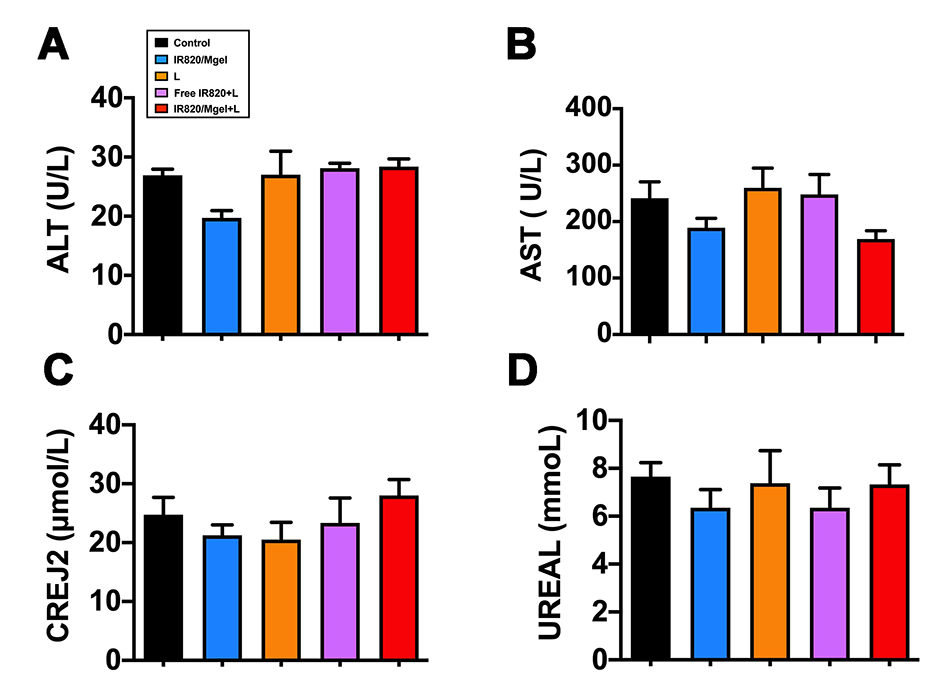

Supplement: Supplementary file 4 — Additional file 4: Figure S4. Blood chemistry analysis of each group. (A) Alanine aminotransferase (ALT); (B) aAspartate aminotransferase (AST); (C) Creatinine (CRE); (D) UREA. [file 12951_2021_1041_MOESM4_ESM.tif]

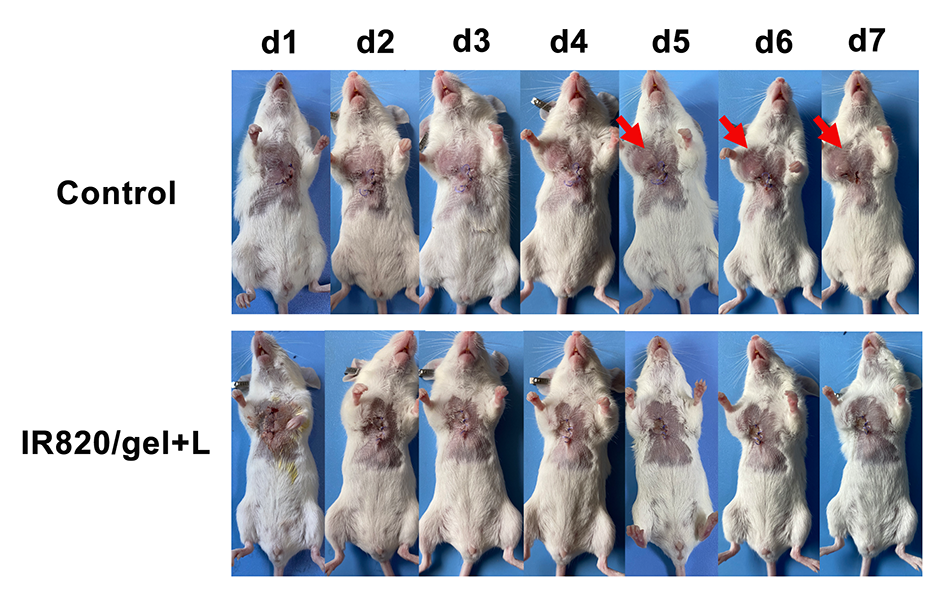

Supplement: Supplementary file 5 — Additional file 5: Figure S5. Phototoxicity of IR820/gel to skin and normal tissues around tumor. [file 12951_2021_1041_MOESM5_ESM.tif]

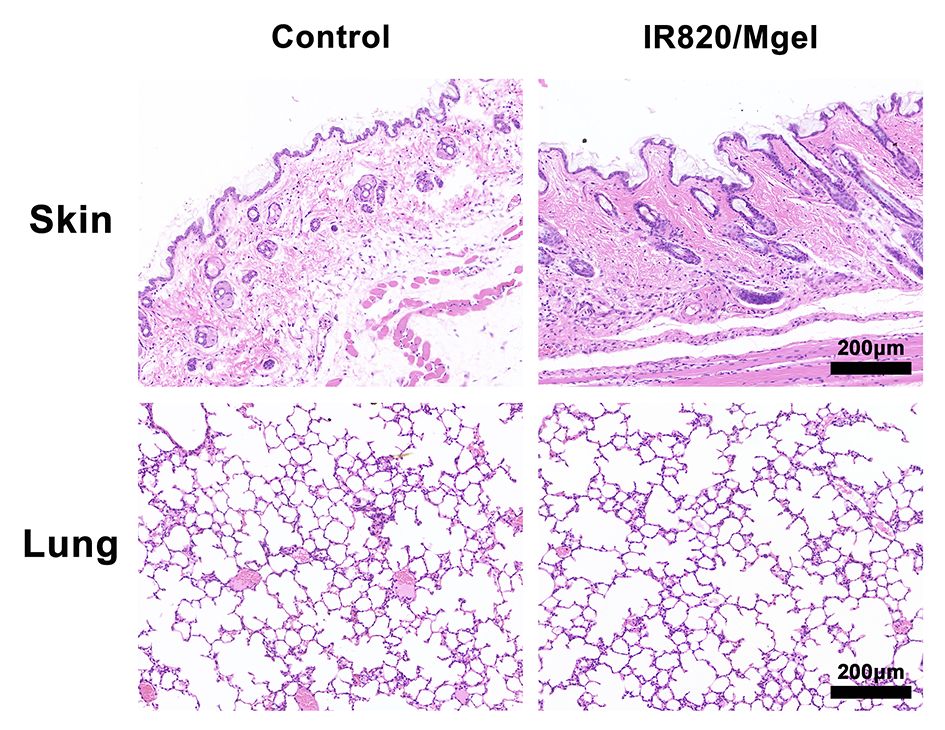

Supplement: Supplementary file 6 — Additional file 6: Figure S6. H&E staining of tissues of incision area and lungs. [file 12951_2021_1041_MOESM6_ESM.tif]

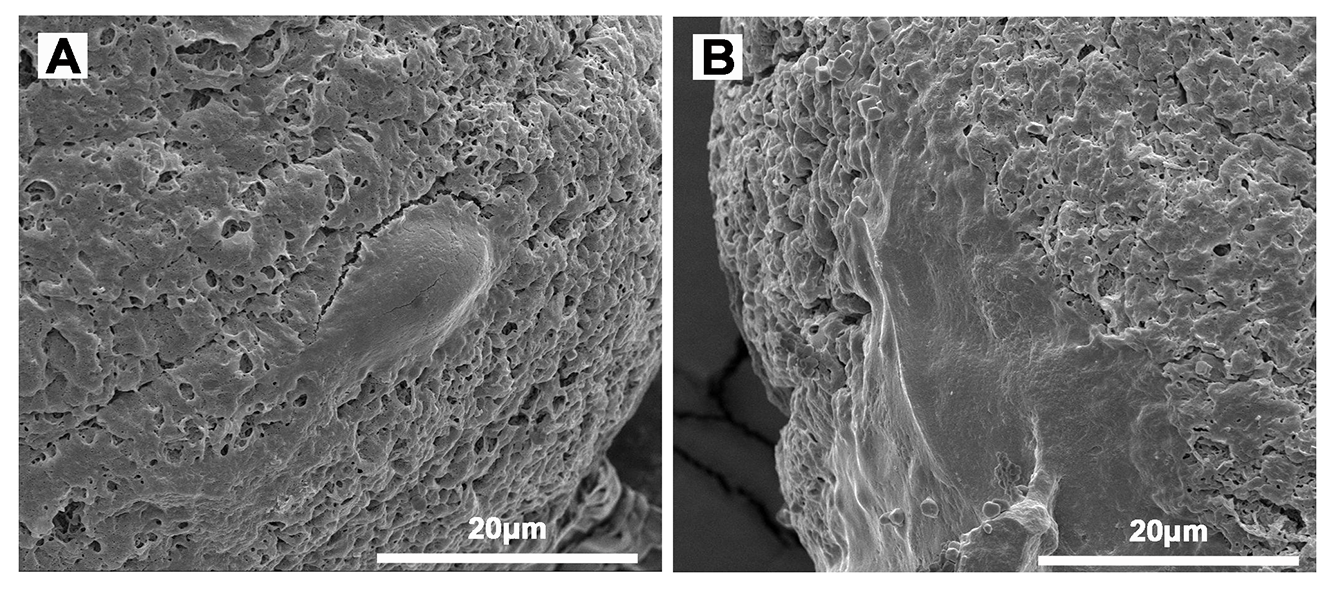

Supplement: Supplementary file 7 — Additional file 7: Figure S7. In vitro breast construction property of hydrogel platform. The SEM micrographs of adipose stem cells adhered porous MPs. [file 12951_2021_1041_MOESM7_ESM.tif]
